# DNA methylation-based detection and prediction of cervical intraepithelial neoplasia grade 3 and invasive cervical cancer with the WID™-qCIN test

**DOI:** 10.1186/s13148-022-01353-0

**Published:** 2022-11-21

**Authors:** Chiara Herzog, Karin Sundström, Allison Jones, Iona Evans, James E. Barrett, Jiangrong Wang, Elisa Redl, Lena Schreiberhuber, Laura Costas, Sonia Paytubi, Lukas Dostalek, Michal Zikan, David Cibula, Gaby Sroczynski, Uwe Siebert, Joakim Dillner, Martin Widschwendter

**Affiliations:** 1 European Translational Oncology Prevention and Screening (EUTOPS) Institute, Universität Innsbruck, Milser Straße 10, 6060 Hall in Tirol, Austria; 2 Research Institute for Biomedical Aging Research, Universität Innsbruck, 6020 Innsbruck, Austria; 3 Department of Laboratory Medicine, Division of Pathology, Karolinska Institutet, Stockholm, Sweden; 4 Medical Diagnostics Karolinska, Karolinska University Hospital, Stockholm, Sweden; 5 Department of Women’s Cancer, UCL EGA Institute for Women’s Health, University College London, 74 Huntley Street, London, WC1E 6AU UK; 6 Cancer Epidemiology Research Programme, Catalan Institute of Oncology. IDIBELL, Av Gran Vía 199-203, 08908 L’Hospitalet de Llobregat, Barcelona, Spain; 7 Consortium for Biomedical Research in Epidemiology and Public Health - CIBERESP, Carlos III Institute of Health, Av. De Monforte de Lemos 5, 28029 Madrid, Spain; 8 Gynaecologic Oncology Center, Department of Obstetrics and Gynecology, First Faculty of Medicine, Charles University in Prague, General University Hospital in Prague, Prague, Czech Republic; 9 Department of Gynecology and Obstetrics, First Faculty of Medicine and Hospital Na Bulovce, Charles University in Prague, Prague, Czech Republic; 10 Institute of Public Health, Medical Decision Making and Health Technology Assessment, Department of Public Health, Health Services Research and Health Technology Assessment, UMIT TIROL – University for Health Sciences and Technology, Hall in Tirol, Austria; 11 Center for Health Decision Science, Boston, MA USA; 12 Department of Epidemiology, Harvard T.H. Chan School of Public Health, Boston, MA USA; 13 Department of Health Policy and Management, Harvard T.H. Chan School of Public Health, Boston, MA USA; 14 Institute for Technology Assessment and Department of Radiology, Massachusetts General Hospital, Harvard Medical School, Boston, MA USA; 15 Department of Women’s and Children’s Health, Karolinska Institutet, Stockholm, Sweden

**Keywords:** DNA methylation, CIN3, HPV, Cervical screening

## Abstract

**Background:**

Cervical screening using primary human papilloma virus (HPV) testing and cytology is being implemented in several countries. Cytology as triage for colposcopy referral suffers from several shortcomings. HPV testing overcomes some of these but lacks specificity in women under 30. Here, we aimed to develop and validate an automatable triage test that is highly sensitive and specific independently of age and sample heterogeneity, and predicts progression to CIN3+ in HPV+ patients.

**Results:**

The WID™-qCIN, assessing three regions in human genes *DPP6*, *RALYL*, and *GSX1,* was validated in both a diagnostic (case–control) and predictive setting (nested case–control), in a total of 761 samples. Using a predefined threshold, the sensitivity of the WID™-qCIN test was 100% and 78% to detect invasive cancer and CIN3, respectively. Sensitivity to detect CIN3+ was 65% and 83% for women < and ≥ 30 years of age. The specificity was 90%. Importantly, the WID™-qCIN test identified 52% of ≥ 30-year-old women with a cytology negative (cyt−) index sample who were diagnosed with CIN3 1–4 years after sample donation.

**Conclusion:**

We identified suitable DNAme regions in an epigenome-wide discovery using HPV+ controls and CIN3+ cases and established the WID™-qCIN, a PCR-based DNAme test. The WID™-qCIN test has a high sensitivity and specificity that may outperform conventional cervical triage tests and can in an objective, cheap, and scalable fashion identify most women with and at risk of (pre-)invasive cervical cancer. However, evaluation was limited to case–control settings and future studies will assess performance and generalisability in a randomised controlled trial.

**Supplementary Information:**

The online version contains supplementary material available at 10.1186/s13148-022-01353-0.

## Background

Cervical cancer is the fourth most common malignancy in women worldwide. In November 2020, the WHO launched an initiative to accelerate the elimination of cervical cancer via vaccination, screening, and treatment [1]. Cervical cancer screening, aiming to identify women with pre-invasive dysplastic lesions which can be surgically excised, is one of the most successful personalised cancer prevention strategies to date [2].

Primary HPV testing has been consistently shown to be superior to other screening methods [3]. As a result, most countries are currently changing screening from primary cytology to primary HPV testing, with cytology as the triage for colposcopic assessment of oncogenic HPV-positive (oncHPV+) women [4]. In Europe, cervical cancer screening participation rates vary between 40.5 and 81.4%, and efforts to increase participation to ≥ 85% are essential. Self-collection of cervicovaginal samples may be more widely acceptable than collection of a cervical screening sample by a healthcare professional (HCP) and offers an alternative option for individuals that may suffer from trauma, embarrassment, or pain, thereby increasing attendance [5]. Self- and HCP-collected sampling shows comparable HPV testing results [6], but cytology is not feasible in self-collected samples and would therefore need to be followed up with a HCP-collected sample. Because less than 60% of women who provide a self-collected sample attend follow-up appointments [7–9] and cytology achieves a sensitivity and specificity for detection of CIN3+ of ~ 80% and only ~ 60% [10–16], respectively, an accurate test to triage oncHPV+ women that could be performed on the same original sample could be highly beneficial to reduce loss-to-triage follow-up and improve detection without false positives.

The feasibility of utilising DNA methylation (DNAme) markers for the detection of pre-invasive or invasive gynaecological cancers has been shown by us [17–20] and others (reviewed in [21, 22]), including in self-collected samples. As outlined in our recent publication [20] describing the use of a methylation array-based signature for detection of cervical malignancies, premalignancies, and risk of malignancy, the clinical use of DNAme markers to triage oncHPV+ women has so far been impeded for several reasons, including a lack of sensitivity for detection of CIN3(+) in women below age 30 who have a high prevalence of oncHPV [23], a lack of assessment of specificity in young women, and the fact that it has largely been unknown whether DNAme markers are able to outperform cytology as an indicator for future disease risk in oncHPV+ women. Our recent work has addressed these issues: a DNA methylation array-based signature, the WID-CIN, is able to identify women with, or at risk of, cervical malignancies, offers high sensitivity and specificity even below the age of 30, and outperforms cytology as an indicator for future disease risk [20]. The advantage of the array-based WID-CIN signature is the ability to detect or predict the risk also for the other women’s cancers using a single cervical sample and one single assay as demonstrated recently for breast, ovarian, and endometrial cancer [24, 25] (latter currently under review).

Despite these clear advantages of an array-based signature, for the current screening which utilises samples collected by health care professionals or self-samples, a simple and accurate PCR-based test is required to triage oncHPV+ women.

Here, we therefore aimed to develop a triage test capable of both detection of current and identification of future CIN3+ and potentially suitable for use with self-collected samples, using a PCR-based approach. Using cervical liquid-based cytology samples in a nested case–control setting for generalisability of our findings, we develop and validate the three-marker PCR-based DNAme WID™-qCIN test for triage of oncHPV+ women.

## Results

### WID™-qCIN test development

**Fig. 1:**
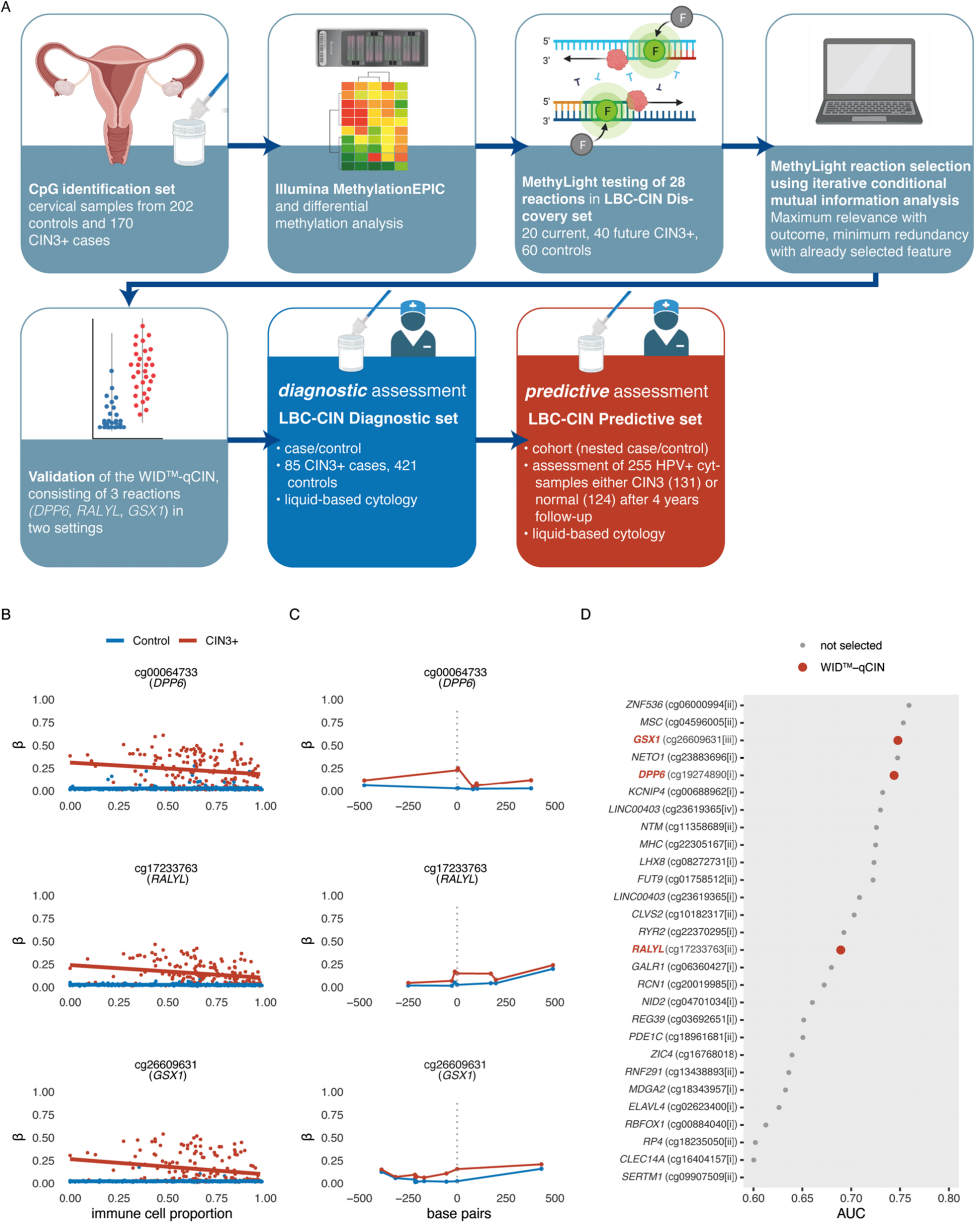
Overview of the study and selection of WID™-qCIN MethyLight reactions. **A** Overview of the study setup. **B** Example plots of CpG methylation beta values in cervical samples of controls and cervical intraepithelial neoplasia grade 3+ (CIN3+) cases versus immune cell proportion (ic) and **C** mean methylation values of CpGs in close genomic proximity (within 500 base pairs) to the index CpG, to identify differentially methylated regions. **D** AUC of individual MethyLight reactions for discrimination of (current or future) CIN3 + cases from controls in the LBC-CIN Discovery set. Three reactions were selected for the WID™-qCIN test using a minimal conditional mutual maximisation filter for feature selection (maximum information with the outcome, minimal redundancy with each other). *CIN* cervical intraepithelial neoplasia, *LBC* liquid-based cytology

An overview of the WID™-qCIN test development is shown in Fig. 1A, with relevant sample sets shown in Additional file 1: Figure S1. To identify suitable regions for MethyLight reactions, we assessed DNA methylation at ~ 850,000 CpG sites in cervical smear samples from women with CIN3+ (*n* = 170) and normal cytology (*n* = 202) (“CpG identification set”) using the Infinium MethylationEPIC array employing a previously established workflow [24]. We designed MethyLight reactions for 28 regions amongst the top 50 differentially methylated positions (DMPs) whose index CpG and surrounding CpGs showed the largest difference between cases and controls and where at least one CpG showed no or very low methylation in controls regardless of cellular composition of the samples (example regions in Fig. 1B, C). We evaluated DNA methylation with MethyLight reactions in 20 current and 40 future cases and 60 controls (LBC-CIN Discovery Set) and ranked reactions according to their area under the receiver operating characteristic curve (AUC of the ROC) (Fig. 1D). We selected the final WID™-qCIN top regions via a mutual information approach [26], aiming to identify those regions which carry maximum information about the outcome (current/future CIN3) but are minimally redundant (i.e. aiming to reduce the number of markers that would only show the same information and therefore not deemed “independent”) (Fig. 1E). This indicated that the combination of three regions in the human genes *DPP6*, *RALYL*, and *GSX1* was most suitable for discriminatory performance. We defined the sum of the three PMR values for *DPP6*, *RALYL*, and *GSX1* (ΣPMR), without any additional weighting, as the WID™-qCIN test. The WID™-qCIN (ΣPMR) led to an AUC of 0.94 for current cases and 0.64 for future cases in the LBC-CIN Discovery Set. Based on prior knowledge from our WID™-qEC test where we identified a threshold using Youden’s index of the AUC [19], we set the test threshold to 0.63 for subsequent diagnostic and predictive validation.

### WID™-qCIN test in cervical smear samples

**Fig. 2:**
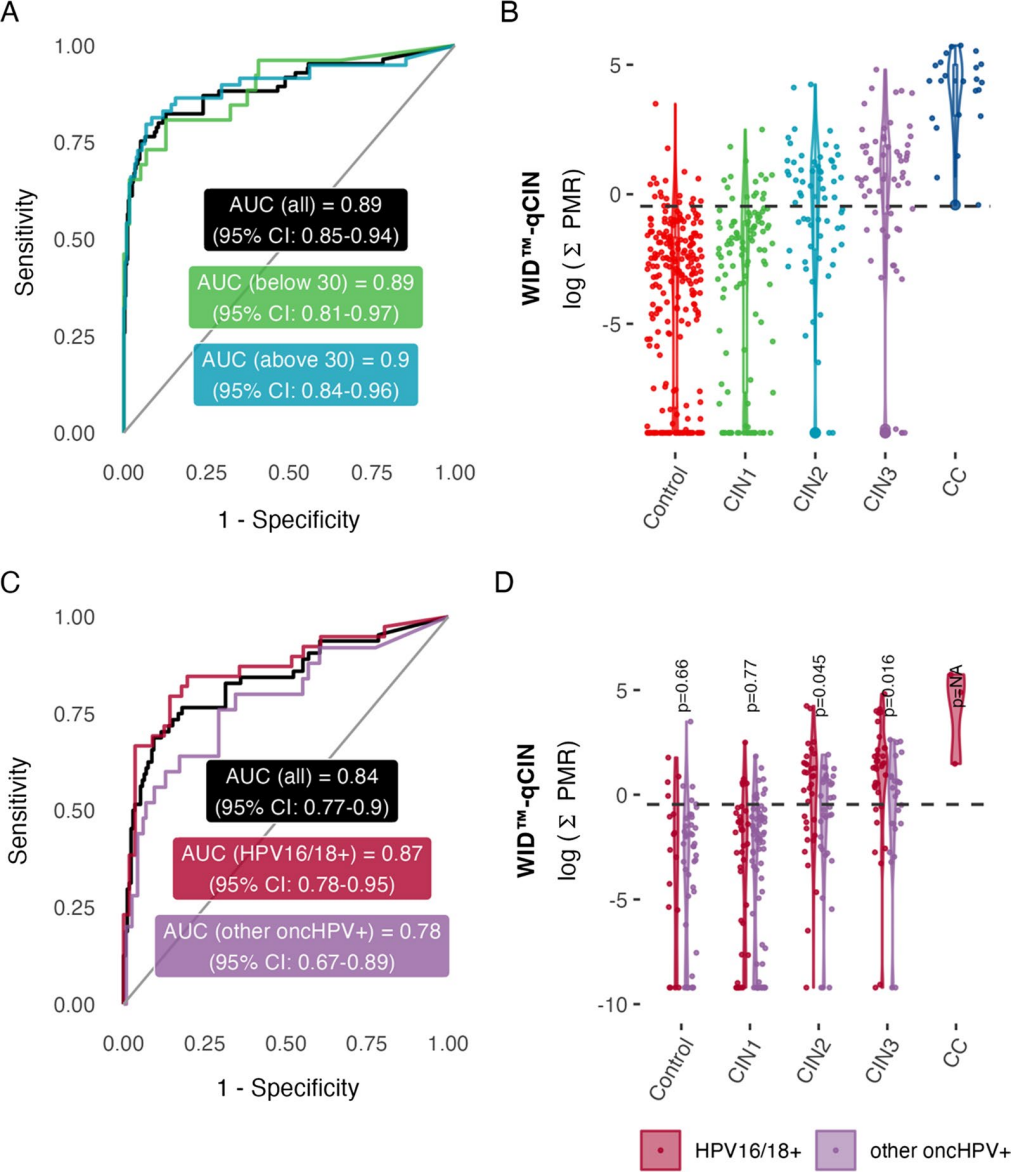
WID™-qCIN test performance in relation to age, diagnosis, and HPV status in the LBC-CIN Diagnostic set.** A** The WID™-qCIN test discriminates well between controls (up to CIN1) and cases (CIN3/AIS and invasive cervical cancers) in the LBC-CIN Diagnostic Validation Set regardless of age, although performance is higher in individuals over 30 years. **B** WID™-qCIN values (log scale) in LBC-CIN Diagnostic set samples. The WID™-qCIN threshold of 0.63 (− 0.46 on a log scale, dashed line) was selected a priori based on prior knowledge. **C** ROC curve analysis of different HPV subtypes (HPV16/18+ vs. other oncHPV+). Curves are not significantly different (DeLong p value = 0.23). **D** The WID™-qCIN is slightly higher in CIN2 samples from HPV16/HPV18+ individuals compared with other oncHPV subtypes (p = 0.04 in Wilcoxon signed-rank test). *AUC* area under the curve, *PMR* percentage methylated reference, *oncHPV* oncogenic human papillomavirus

Validation of the WID™-qCIN test in 506 cervical smear samples in the LBC-CIN Diagnostic Set (Table 1) with CIN3 + as the outcome resulted in an AUC of 0.89, comparing CIN3+ versus ≤ CIN1. Stratification by ages ≥ 30 and < 30 years led to AUCs of 0.9 and 0.89, respectively (Fig. 2A). The WID™-qCIN ΣPMR values of the LBC-CIN Diagnostic Set are displayed on a log scale in Fig. 2B for illustrative purposes. (Additional file 1: Figure S2 shows values of individual regions.) WID™-qCIN identification of CIN3+ cases from controls and CIN1 cases was not dependent on HPV subtype (Fig. 2C), although identification was slightly superior in samples from women positive HPV16 or HPV18+ compared to other oncogenic HPV (oncHPV) subtypes (Fig. 2D). HPV16/18+ CIN3 cases had a small but significant increase in the WID™-qCIN compared with other oncHPV+ CIN3 cases.

**Table 1:** Overview of datasets

Characteristic	Diagnostic validation		Predictive validation	
	LBC-CIN Diagnostic		LBC-CIN Predictive	
	Control *n* = 355	Case *n* = 151	Control *n* = 124	Case *n* = 131
Age	33 (27–39)	33 (27–40)	31 (26–38)	30 (26–37)
Setting, *n* (%)	Case/control		Cohort (nested case/control)	
HPV status				
HPV16/18+	56 (16)	72 (48)	30 (24)	73 (56)
other oncHPV+	116 (33)	58 (38)	94 (76)	58 (44)
oncHPV−	154 (43)	1 (0.7)		
Unknown	29 (8.2)	20 (13)		
Diagnosis				
Normal cytology	239 (67)		124 (100)	
CIN1	116 (33)			
CIN2		66 (44)		
CIN3		62 (41)		131 (100)
Invasive cervical cancer		23 (15)		
Time to event (years)			3.14 (3.03–3.30)	3.11 (2.66–3.25)

Numbers in brackets indicate interquartile range or percentage for continuous and categorical variables, respectively

*oncHPV* high-risk human papillomavirus, *CIN* cervical intraepithelial neoplasia, *AIS* adenocarcinoma in situ

Applying the pre-specified threshold of 0.63 led to a 14%, 55%, 69%, and 100% sensitivity for the detection of CIN1, CIN2, CIN3, and invasive cervical cancers, respectively (Additional file 1: Table S1). Importantly, the performance was highly similar when restricting to HPV+ samples only (Additional file 1: Table S2). The sensitivity for CIN3+ detection in the LBC-CIN Diagnostic Set was 78%, with 83% and 65% in women ≥ 30 and < 30 years, respectively (Table 2). Importantly, this outperformed HPV typing, which showed generally lower combined sensitivity and specificities (Additional file 1: Table S3).

**Table 2:** Sensitivity and specificity of the WID™-qCIN test

	Diagnostic validation	Predictive validation
	LBC-CIN Diagnostic	LBC-CIN Predictive
All, % (95% CI)		
Specificity (≤ CIN1)	90 (87–93)	93 (87–97)
Sensitivity (CIN2)	55 (42–67)	–
Sensitivity (CIN3)	69 (56–80)	35 (27–44)
Sensitivity (CIN3+)	78 (67–86)	35 (27–44)
Sensitivity (CC)	100 (85–100)	–
PPV (CIN3+)	59 (52–68)	57 (43–72)
NPV (≤ CIN1)	93 (91–95)	84 (82–86)
NPV (≤ CIN2)	92 (90–95)	84 (82–86)
Age < 30 years, % (95% CI)		
Specificity (≤ CIN1)	95 (89–98)	92 (82–97)
Sensitivity (CIN2)	46 (28–66)	–
Sensitivity (CIN3)	59 (36–79)	15 (7–27)
Sensitivity (CIN3+)	65 (44–83)	15 (7–27)
Sensitivity (CC)	100 (40–100)	–
PPV (CIN3+)	76 (55–91)	33 (17–58)
NPV (≤ CIN1)	91 (86–94)	80 (78–82)
NPV (≤ CIN2)	89 (84–94)	80 (78–82)
Age ≥ 30 years, % (95% CI)		
Specificity (≤ CIN1)	88 (83–92)	94 (85–98)
Sensitivity (CIN2)	61 (43–76)	–
Sensitivity (CIN3)	75 (59–87)	52 (40–64)
Sensitivity (CIN3+)	83 (71–92)	52 (40–64)
Sensitivity (CC)	100 (82–100)	–
PPV (CIN3+)	57 (49–65)	70 (50–86)
NPV (≤ CIN1)	95 (92–97)	88 (85–90)
NPV (≤ CIN2)	94 (91–97)	88 (85–90)

We assessed the WID™-qCIN test in controls (control and CIN1) for different stages of CIN (CIN2, CIN3, CC) in different age groups and datasets as well as in relation to other gynaecological diseases (endometrial and ovarian cancer). For assessment of positive and negative predictive values (PPV, NPV), we included only HPV+ individuals and assumed a prevalence of 21.5% in line with results from a previous systematic review reporting CIN3+ prevalence in triaged women [21]. NPV is reported separately when including samples ≤ CIN1 or ≤ CIN2 as controls

*CIN* cervical intraepithelial neoplasia, *CC* cervical cancer (includes adenocarcinoma in situ and invasive cervical cancer), *PPV* positive predictive value, *NPV* negative predictive value

The specificity, based on HPV+ and HPV−/cyt− samples as well as HPV+/cyt+ samples which were diagnosed as CIN1 on histology, was 90% in the LBC-CIN Diagnostic Set, and 95% and 88% in women ≥ and < 30 years of age, respectively. Importantly, the WID™-qCIN test only deemed 14% overall women with CIN1, and 17% and 8% of women ≥ 30 and < 30 years, respectively, as positive (Additional file 1: Table S1).

Assuming a CIN3+ population prevalence of 21.5% in pre-screened women (e.g. HPV+ individuals), as previously reported [21], the positive (PPV) and negative (NPV) predictive values for > CIN3+ and ≤ CIN1, respectively, in the LBC-CIN Diagnostic Set in all HPV+ women, regardless of age, were 59% and 93% (Table 2). The PPV and NPV (CIN3+ and ≤ CIN1) in women ≥ 30 years of age in this set were 57% and 95%, while they were 76% and 91% in women < 30 years, respectively (Table 2). Inclusion of CIN2 for computation of NPV (≤ CIN2) did not alter values much, although this will need to be further validated as the number of CIN2 samples in our set was relatively small (*n* = 66).

### WID™-qCIN to predict future cancer risk

To interpret a positive test in the absence of current cervical pathologies, we furthermore aimed to assess whether the WID™-qCIN test could identify future disease in HPV+/cyt− women donating samples 1–4 years in advance of a CIN3 diagnosis.

**Fig. 3:**
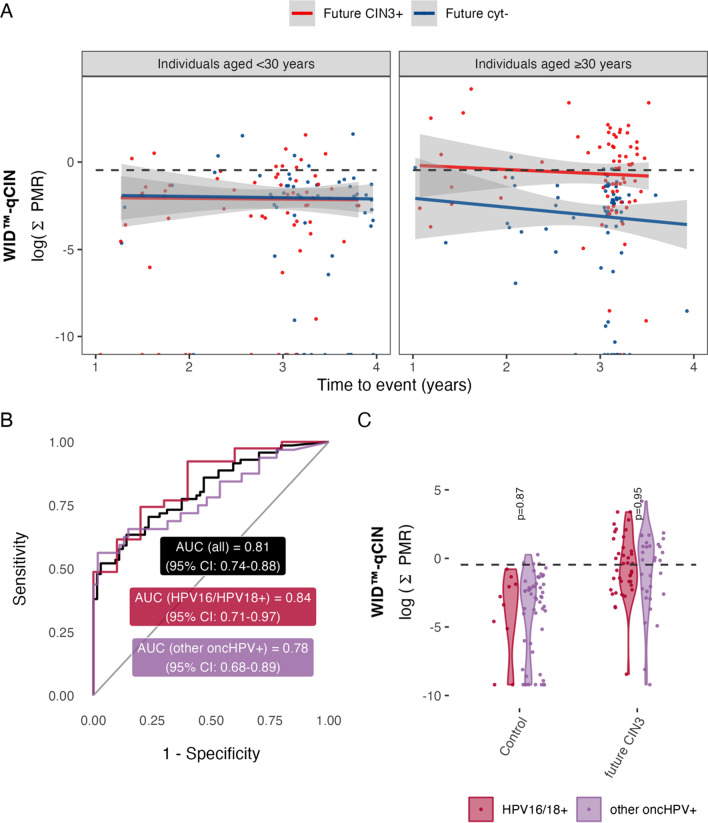
Influence of time to future CIN3 on WID™-qCIN in individuals below and above 30 years of age and in relation to HPV status in the LBC-CIN Predictive set. **A** Scatter plot of time to event (censoring/future cytology negative (cyt−) or CIN3+ diagnosis) versus logarithm of the raw PMR values of the WID™-qCIN. PMR values are visualised on log scale only for illustrative purposes. The dashed line indicates the threshold [log(0.63) = − 0.46]. **B** AUC of the WID™-qCIN stratified by HPV16/18+ or other oncHPV+ status. Curves are not significantly different (DeLong p value = 0.52). **C** WID™-qCIN values in HPV16/HPV18+ or other oncHPV+ individuals and future CIN3 cases. *CIN3* cervical intraepithelial neoplasia grade 3, *cyt−* cytology negative, *AUC* area under the curve, *PMR* percentage methylated reference, *oncHPV* oncogenic human papillomavirus

Amongst HPV+/cyt− women aged ≥ 30 and < 30 years diagnosed with CIN3 1–4 years after sample donation, 52% and 15% were WID™-qCIN positive, respectively (Table 2). The specificity for women ≥ 30 and < 30 years of age was 94% and 92%, respectively (Table 2, Fig. 3). WID™-qCIN values were not significantly different between HPV16/18+ individuals and other oncHPV+ individuals who were either future cyt− or CIN3+ (Fig. 3B, C).

## Discussion

Here, we demonstrate that a PCR-based DNA methylation signature, the WID™-qCIN test, may outperform cytology as a triage. Importantly, in addition to high sensitivity and specificity for current cases, the test is also able to identify future cases: 52% and 15% of HPV+/cyt− women ≥ 30 and < 30 years of age, respectively, diagnosed with CIN3 1–4 years after index sample donation, had a positive WID™-qCIN. While a negative cytology followed by a positive diagnosis 1–2 years later could be interpreted as issues with cytological sampling or classification at the initial visit, we argue that this is an inherent weakness of cytology as a standard of care, and sensitivity for current or future disease could be improved with the WID™-qCIN. The WID™-qCIN test also offers high specificity and only identifies 16% and 8% of HPV+/cyt+ women aged ≥ 30 and < 30 years, respectively, who eventually only show CIN1 on biopsy and are therefore deemed to be false cyt+ (Additional file 1: Table S2).

Only a small number of studies assessed the clinical validity of DNAme markers in cervical liquid-based cytology samples in a primary screening setting, as summarised in our recent publication [20]. Briefly, a prospective study by Verhoef et al., evaluating the use of DNAme in *MAL* and *miR-124-2*, reported a lower sensitivity of DNAme than cytology triage (67.5% versus 74.8%, respectively), required twice as many colposcopy referrals, and only included women ≥ 33 years [11]. The performance of the methylation markers described by Verhoef et al. may presumably have been worse in younger women [27]. Although we also observed an age-dependent performance of the WID™-qCIN test, we were able to achieve a sensitivity of 65% at a 95% specificity in this age group in women < 30 years of age (Table 2).

Although tests were not carried out side by side in the same cohort, when comparing sensitivities and specificities of the new WID™-qCIN test with *QIAsure*, a commercially available DNAme test, the WID™-qCIN test exhibits an improved performance (overall sensitivity and specificity = 77 and 91 vs. 77.2 and 78.3). Of note, the majority of women in a recent *QIAsure* set were aged ≥ 29 years [28], with a mean age of 40.7 years, and all CIN/HPV tests to date perform substantially better in older women. Conversely, the mean age in our dataset was 33.7 years. In addition, DNAme of *FAM19A4,* a gene which we described first to become aberrantly methylated in cervical carcinogenesis [29] and which is one of the two regions assessed in the *QIAsure* test, was not amongst the top differentially methylated regions.

For payers and health policy decision makers in countries such as Austria and possibly in other European countries with similar standards for primary cervical cancer screening, long-term modelling results (Additional file 1) highlight that utilising WID™-qCIN testing at three yearly intervals might be an effective and likely cost-effective cervical cancer even as a primary screening modality, although this is not the envisioned initial use of the WID™-qCIN test, which is initially designed as a triage test following HPV screening. As for all model-based studies, our analysis has potential limitations based on the assumptions made and data used. One limitation is that effectiveness data were based on different evidence levels. We used test accuracy data from international meta-analyses of randomised screening trials for cytology and HPV-based screening, whereas test accuracy of the WID™-qCIN test was derived from original data based on a case–control setting, and therefore, incremental cost-effectiveness results may be biased and have limited external validity. However, our sensitivity analyses showed that results are mostly robust when varying assumptions. Further independent research on test accuracy may further reduce uncertainty.

The strengths of this study include the use of only samples from a well-defined population-based screening cohort under careful design to control for potential bias due to factors such as age, sample year, and time of storage, with a comprehensive registry linkage strategy that allowed for the identification of samples long preceding disease. In addition, we employed an epigenome-wide array-based approach for de novo identifying the most informative CpG sites in order to identify women with or at future risk of CIN3+ diagnosis and used a different modality (PCR) to validate the signature.

In summary, in addition to a recent array-based WID-CIN signature for detection and risk prediction of cervical cancer [20], here we have demonstrated the performance of the three-marker PCR-based DNA methylation WID™-qCIN test in triaging women with or at future risk of CIN3+ diagnosis. Whether the array-based WID-CIN or PCR-based WID™-qCIN should be utilised may depend on the setting and aim of screening. Our recent report on the feasibility of the use of cervicovaginal self-samples using MethyLight-based testing for endometrial cancer detection suggests that self-collected samples may also be suitable for the WID™-qCIN test, although this will need to be further validated in individual studies. A strength of this approach is that HPV+ self-collected samples could be rapidly followed up using an automatable platform, making use of the same original sample without the need for patient recall and repeated sample testing. Taken together, our data indicate that the WID™-qCIN test may represent a promising triage strategy for cervical cancer screening and may be prioritised for comprehensive cost-effectiveness analyses and potentially rapid implementation in the clinical arena.

## Methods

### Cervical liquid-based cytology sample collection

All cervical liquid-based cytology samples processed in the capital region of Stockholm in Sweden are biobanked through a state-of-the-art platform at the Karolinska University Laboratory, Karolinska University Hospital, as previously described [30]. Since the year 2013, virtually all of the ~ 150,000 LBCs per year are compacted and stored in a 600 µl, 96 well plate format at − 27 °C. This allows for preservation of intact cells and subsequent analyses of DNA, RNA, and protein content, among others. The biobank is linked to the Swedish health register infrastructure for cytology/HPV results, histopathology test and results, as well as cervical cancer diagnoses, through the individually unique personal identification number (PIN) [31]. We defined cohorts of women resident in Stockholm (Additional file 1: Figure S2), participating in cervical screening, or clinically indicated testing during the years 2013–2017, and have screening sample(s) stored in the biobank. An overview of the Swedish cervical cancer screening programme at the time of the sample collection for this study is shown in Additional file 1: Figure S3. We linked them to the National Cancer Register at the Swedish National Board of Health and Welfare, and the Swedish National Cervical Screening Registry, to identify all current or future cases of CIN3/Adenocarcinoma in situ (AIS) or invasive cervical cancer (CIN3+) and defined datasets for discovery and diagnostic and predictive testing of the WID™-qCIN test.

#### Discovery (*n* = 465)

*CpG Identification set.* For epigenome-wide assessment of cervical cancer markers, we utilised cervical samples from 202 HPV+ cyt− women and 170 women with CIN3+ part of the LBC-CIN Discovery and Diagnostic Sets.

*LBC-CIN Discovery set.* The LBC-CIN Discovery set consisted of 20 samples from current CIN3 + cases and 20 age-matched controls as well as 40 samples of future CIN3+ cases and 40 age-matched controls, i.e. subsets selected from samples above. Samples with sufficient DNA were included and current/future CIN3+ cases were selected.

#### Diagnostic validation (*n* = 506)

*LBC-CIN Diagnostic set.* All screening-derived samples that were cytology-positive during 1–90 days prior to CIN3+ diagnoses in 2013–2015 were defined as cases. Controls were randomly selected from samples that were cyt- in women having no historical cervical lesions and frequency matched (to CIN3+) 1:1 on age group and calendar year of samples. We also identified samples during 1–90 days prior to histologically diagnosed CIN1 and CIN2 with similar age distribution to assess the discrimination ability to exclude low-risk lesions.

#### Predictive validation (*n* = 255)

*LBC-CIN Predictive set.* For assessment of CIN3+ prediction, all cervical samples that were oncHPV+ and cyt− during 2014–2016 from women who were future diagnosed with CIN3+ up to the end of 2017 were defined as cases. The vast majority of future case samples were collected in 2014 (515 out of 669, 77%), the year Stockholm county initiated randomised healthcare policy trial for primary HPV testing. Random oncHPV+ and cyt− samples of women who did not have CIN3+ diagnosis up to the end of 2017 were selected as controls, frequency matched 1:1 on age group, calendar year and type of samples (screening or clinically indicated). All women tested oncHPV+ and cyt− were recalled after 3 years, and 85% attended the follow-up in the recall. All samples for which no HPV results were available were put through high-performance HPV testing on the cobas® 4800 assay [32].

An overview of all datasets and corresponding characteristics is shown in Table 1 and Fig. 1.

### WID™-qCIN assay development

The WID™-qCIN test is based on data from ~ 850,000 methylation sites from 202 HPV+/cyt− women and 170 women with CIN3+ generated on the Illumina MethylationEPIC array platform which indicated CpGs of interest in an epigenome-wide manner. For development of the WID™-qCIN PCR-based assay, we assessed the top 50 differentially methylated positions between women with CIN3+ and those without, including regions 500 bp up- or downstream of the site of interest. Following visual inspection, 28 suitable regions, i.e. those who showed a methylation of 0 (or near 0) in controls and increased methylation in CIN3+ cases across several adjacent CpGs, were selected for development of MethyLight reactions (three exemplary regions shown in Fig. 1B). To account for cellular heterogeneity in cervical samples that consist of both epithelial and immune cells, we plotted exemplary regions against inferred immune cell proportion and verified that methylation differences were present across different sample compositions. These reactions were tested in the LBC-CIN Discovery Set (see Additional file 1: Figure S1) consisting of samples from current CIN3 cases (*n* = 20), controls (*n* = 20), and future CIN3 cases with matched controls (*n* = 40 each). Three reactions were selected for the WID™-qCIN test using minimal conditional mutual information maximisation (CMIM) [26], aimed at identifying those features which are maximally relevant with the output (diagnosis) but minimally redundant with each other in order maximise the information obtained from three combined regions.

### WID™-qCIN DNA methylation assay

DNA methylation-specific, quantitative real-time PCR (MethyLight) analysis was performed as previously described [33] with some modifications. Cervical DNA was extracted and normalised to 25 ng/μl using the Nucleo-Mag Blood 200 µl kit (Macherey Nagel, cat #744501.4). DNA concentration was measured using the Qubit™ 4 Fluorometer (Invitrogen™). 500 ng of extracted DNA was bisulphite modified and eluted to a concentration of 4 ng/µl using the EZ-96 DNA Methylation-Lightning™ Kit (Zymo Research corp, cat. #D5033) as per the manufacturer’s protocol. For the multiplex MethyLight assay, 20 ng of bisulphite modified DNA was amplified in a 20 µl reaction containing 1× Luna® Universal Probe qPCR Master Mix (NEB®, cat. #M3004G) and one of the primer–probe sets listed in Additional file 1: Table S4. All PCR reactions were run in duplicates. To normalise for DNA input in each reaction, *COL2A1* was selected as the reference gene. Human SssI-treated DNA or double-stranded gBlocks™ Gene Fragments (IDT™) containing known copy-numbers of each analysed target and *COL2A1* functioned as equivalent fully methylated calibrator and as qPCR standard curve material. PCR reactions were run on the QuantStudio™ 7 Pro (Applied Biosystems™) and results further extracted via the Design & Analysis Software 2.5.0 (Applied Biosystems™). The percentage of fully methylated reference (PMR) molecules at the target locus was standardised using an R script, dividing the *TARGET*:*COL2A1* input amount ratio (derived using the *COL2A1* standard curve; Eq. 1) of a sample by the *TARGET*:*COL2A1* input amount ratio of gBlocks™ Gene Fragments DNA and multiplying by 100 (Eq. 2).

1 $$\text{input amount}={10}^{\frac{\text{Ct target}-\text{intercept}[\text{COL}2A1 \text{standard curve}]}{\text{slope}[\text{COL}2A1 \text{standard curve}]}}$$

2 $$\text{PMR}= \frac{\frac{\text{input amount target}}{\text{input amount COL}2A1}[\text{Sample}]}{\frac{\text{input amount target}}{\text{input amount COL}2A1}[\text{gBlock}]} \times 100$$

### Statistical analyses

All statistical analyses were conducted using R version 4.0.2 (2020-06-22). Cellular composition of cervical samples analysed using the Illumina MethylationEPIC array was inferred using the EpiDISH algorithm, version 2.10.0 [34]. Three regions were selected using the minimal conditional mutual information maximisation filter function (CMIM) in the praznik package, version 9.0.0, based on the method developed by Fleuret [26]. Receiver operating characteristic curves, areas under the curve, and corresponding 95% confidence intervals were generated using the pROC package, version 1.18.0. Sensitivity and specificity including 95% confidence intervals were calculated using the epi.tests function in the epiR package, version 2.0.38, while PPV and NPV were obtained from the BDtest function (bdpv package, version 1.3) assuming a CIN3+ prevalence of 21.5% for pre-screened individuals (i.e. HPV+ individuals) following a previous systematic review for CIN3+ [21]. All statistics were conducted on original ∑PMR values. Original data are available in Additional file 2, Tables 1, 2 and 3.

**Table 3:** Diagnostic and predictive WID™-qCIN assessment in distinct HPV subgroups

	LBC-CIN diagnostic		LBC-CIN predictive	
	HPV16/HPV18+	Other oncHPV+	HPV16/HPV18+	Other oncHPV+
All, % (95% CI)				
*n* (controls, cases)	56, 39	116, 25	30, 73	94, 58
Sensitivity (CIN3+)—% (95% CI)	79 (64–91)	56 (35–76)	34 (24–46)	36 (24–50)
Specificity (≥ CIN1)—% (95% CI)	84 (72–92)	87 (80–93)	90 (73–98)	94 (87–98)
PPV—% (95% CI)^†^	58 (45–72)	54 (42–68)	48 (27–74)	61 (43–78)
NPV—% (95% CI)^†^	94 (90–97)	88 (83–92)	83 (81–86)	84 (82–87)
Age < 30 years, % (95% CI)				
*n* (controls, cases)	12, 14	37, 9	20, 34	40, 26
Sensitivity (CIN3+)—% (95% CI)	79 (49–95)	33 (7–70)	18 (7–35)	12 (2–30)
Specificity (≥ CIN1)—% (95% CI)	92 (62–100)	95 (82–99)	85 (62–97)	95 (83–99)
PPV—% (95% CI)^†^	72 (34–95)	63 (30–90)	24 (10–53)	39 (13–78)
NPV—% (95% CI)^†^	94 (87–98)	84 (78–89)	79 (75–83)	80 (77–82)
Age ≥ 30 years, % (95% CI)				
*n* (controls, cases)	44, 25	79, 16	10, 39	54, 32
Sensitivity (CIN3+)—% (95% CI)	80 (59–93)	69 (41–89)	49 (32–65)	56 (38–74)
Specificity (≥ CIN1)—% (95% CI)	82 (67–92)	84 (74–91)	100 (69–100)	93 (82–98)
PPV—% (95% CI)^†^	55 (41–70)	53 (41–68)	56 (24–79)	68 (48–85)
NPV—% (95% CI)^†^	94 (88–97)	91 (84–95)	86 (83–90)	89 (85–92)

The WID™-qCIN test was assessed in individuals either positive for HPV16/18 (HPV1618+) or other oncogenic human papillomaviruses (oncHPV+), looking at both current (LBC-CIN Diagnostic) or future (LBC-CIN Predictive) cases. Results are also provided stratified by age group

*PPV* positive predictive value, *NPV* negative predictive value

^†^Assumed population prevalence in the above sets: 21.5%

## Supplementary Information

**Additional file 1:** Additional figures and tables.

**Additional file 2:** Original data for calculations in the manuscript.

## Data Availability

The epigenome-wide dataset supporting the conclusions of this article is available in the European Genome-Phenome-Archive repository with the unique identifier EGAS00001005078. The PCR-based DNAme datasets supporting the conclusions of this article are included within the article and its additional files.

## Abbreviations

ASCUS: Atypical squamous cells of undetermined significance
AUC: Area under the (receiver operating characteristic) curve
CIN(1–3+): Cervical intraepithelial neoplasia, grade (1–3+)
CMIM: Conditional mutual information maximisation
cyt+: Cytology positive
cyt−: Cytology negative
DNAme: DNA methylation
DMP: Differentially methylated position
FIGO: International Federation of Gynecology and Obstetrics
GDP: Gross domestic product
HPV: Human papillomavirus
HPV+: Human papillomavirus positive
HPV−: Human papillomavirus negative
HPV16: Human papillomavirus subtype 16
HPV18: Human papillomavirus subtype 18
oncHPV: Oncogenic human papillomavirus
oncHPV+: Oncogenic human papillomavirus positive
oncHPV−: Oncogenic human papillomavirus negative
ICER: Incremental cost-effectiveness ratio
LBC: Liquid-based cytology
LBC-CIN: Liquid-based cytology intraepithelial neoplasia
LSIL: Low-grade squamous intraepithelial lesion
NPV: Negative predictive value
Pap: Papanicolaou test
PCR: Polymerase chain reaction
PMR: Percentage methylated reference
PPV: Positive predictive value

## References

[1] Das M (2020). WHO launches strategy to accelerate elimination of cervical cancer. Lancet Oncol.

[2] Cohen PA, Jhingran A, Oaknin A, Denny L (2019). Cervical cancer. Lancet.

[3] Bouvard V, Wentzensen N, Mackie A, Berkhof J, Brotherton J, Giorgi-Rossi P (2021). The IARC perspective on cervical cancer screening. N Engl J Med.

[4] Force USPST, Curry SJ, Krist AH, Owens DK, Barry MJ, Caughey AB (2018). Screening for cervical cancer: US preventive services task force recommendation statement. JAMA.

[5] Nelson EJ, Maynard BR, Loux T, Fatla J, Gordon R, Arnold LD (2017). The acceptability of self-sampled screening for HPV DNA: a systematic review and meta-analysis. Sex Transm Infect..

[6] Polman NJ, Ebisch RMF, Heideman DAM, Melchers WJG, Bekkers RLM, Molijn AC (2019). Performance of human papillomavirus testing on self-collected versus clinician-collected samples for the detection of cervical intraepithelial neoplasia of grade 2 or worse: a randomised, paired screen-positive, non-inferiority trial. Lancet Oncol.

[7] Sancho-Garnier H, Tamalet C, Halfon P, Leandri FX, Retraite LL, Djoufelkit K (2013). HPV self-sampling or the Pap-smear: a randomized study among cervical screening nonattenders from lower socioeconomic groups in France. Int J Cancer.

[8] Broberg G, Gyrd-Hansen D, Jonasson JM, Ryd ML, Holtenman M, Milsom I (2014). Increasing participation in cervical cancer screening: offering a HPV self-test to long-term non-attendees as part of RACOMIP, a Swedish randomized controlled trial. Int J Cancer.

[9] Cadman L, Wilkes S, Mansour D, Austin J, Ashdown-Barr L, Edwards R (2015). A randomized controlled trial in non-responders from Newcastle upon Tyne invited to return a self-sample for Human Papillomavirus testing versus repeat invitation for cervical screening. J Med Screen.

[10] Bosgraaf RP, Verhoef VMJ, Massuger LFAG, Siebers AG, Bulten J, Ridder GMDK (2015). Comparative performance of novel self-sampling methods in detecting high-risk human papillomavirus in 30,130 women not attending cervical screening. Int J Cancer.

[11] Verhoef VM, Bosgraaf RP, van Kemenade FJ, Rozendaal L, Heideman DA, Hesselink AT (2014). Triage by methylation-marker testing versus cytology in women who test HPV-positive on self-collected cervicovaginal specimens (PROHTECT-3): a randomised controlled non-inferiority trial. Lancet Oncol.

[12] Wentzensen N, Fetterman B, Castle PE, Schiffman M, Wood SN, Stiemerling E (2015). p16/Ki-67 dual stain cytology for detection of cervical precancer in HPV-positive women. JNCI J National Cancer Inst..

[13] Bergeron C, Giorgi-Rossi P, Cas F, Schiboni ML, Ghiringhello B, Palma PD (2015). Informed cytology for triaging HPV-positive women: substudy nested in the NTCC randomized controlled trial. JNCI J National Cancer Inst..

[14] Luttmer R, Strooper LMAD, Berkhof J, Snijders PJF, Dijkstra MG, Uijterwaal MH (2016). Comparing the performance of FAM19A4 methylation analysis, cytology and HPV16/18 genotyping for the detection of cervical (pre)cancer in high-risk HPV-positive women of a gynecologic outpatient population (COMETH study). Int J Cancer.

[15] Richardson LA, El-Zein M, Ramanakumar AV, Ratnam S, Sangwa-Lugoma G, Longatto-Filho A (2015). HPV DNA testing with cytology triage in cervical cancer screening: influence of revealing HPV infection status. Cancer Cytopathol.

[16] Wright TC, Stoler MH, Aslam S, Behrens CM (2016). Knowledge of patients’ human papillomavirus status at the time of cytologic review significantly affects the performance of cervical cytology in the ATHENA study. Am J Clin Pathol.

[17] Widschwendter A, Gattringer C, Ivarsson L, Fiegl H, Schneitter A, Ramoni A (2004). Analysis of aberrant DNA methylation and human papillomavirus DNA in cervicovaginal specimens to detect invasive cervical cancer and its precursors. Clin Cancer Res.

[18] Doufekas K, Zheng SC, Ghazali S, Wong M, Mohamed Y, Jones A, et al. DNA methylation signatures in vaginal fluid samples for detection of cervical and endometrial cancer. Int J Gynecol Cancer. 2016; Available from: https://www.ncbi.nlm.nih.gov/pubmed/27258725 10.1097/IGC.0000000000000739 27258725

[19] Herzog C, Marín F, Jones A, Evans I, Reisel D, Redl E, et al. A simple cervicovaginal epigenetic test for screening and rapid triage of women with suspected endometrial cancer: validation in several cohort and case/control sets. J Clin Oncol. 2022. 10.1200/JCO.22.00266 PMC9671754 36001862

[20] Barrett JE, Sundström K, Jones A, Evans I, Wang J, Herzog C (2022). The WID-CIN test identifies women with, and at risk of, cervical intraepithelial neoplasia grade 3 and invasive cervical cancer. Genome Med.

[21] Kelly H, Benavente Y, Pavon MA, Sanjose SD, Mayaud P, Lorincz AT (2019). Performance of DNA methylation assays for detection of high-grade cervical intraepithelial neoplasia (CIN2+): a systematic review and meta-analysis. Br J Cancer.

[22] Kremer W, Steenbergen R, Heideman D, Kenter G, Meijer C (2021). The use of host cell DNA methylation analysis in the detection and management of women with advanced cervical intraepithelial neoplasia: a review. BJOG.

[23] Bruni L, Diaz M, Castellsague X, Ferrer E, Bosch FX, de Sanjose S (2010). Cervical human papillomavirus prevalence in 5 continents: meta-analysis of 1 million women with normal cytological findings. J Infect Dis.

[24] Barrett JE, Herzog C, Jones A, Leavy OC, Evans I, Knapp S (2022). The WID-BC-index identifies women with primary poor prognostic breast cancer based on DNA methylation in cervical samples. Nat Commun.

[25] Barrett JE, Jones A, Evans I, Reisel D, Herzog C, Chindera K (2022). The DNA methylome of cervical cells can predict the presence of ovarian cancer. Nat Commun.

[26] Fleuret F (2004). Fast binary feature selection with conditional mutual information. J Mach Learn Res.

[27] Beiersdorf J, Scheungraber C, Wunsch K, Schmitz M, Hansel A, Hoyer H (2020). Combined assessment of 3q26 amplification and promoter methylation in patients with high grade cervical lesions show age specific differences. Genes Chromosomes Cancer.

[28] Bonde J, Floore A, Ejegod D, Vink FJ, Hesselink A, van de Ven PM, et al. Methylation markers FAM19A4 and miR124-2 as triage strategy for primary HPV screen positive women; a large European multi-center study. Int J Cancer. 2020; Available from: https://www.ncbi.nlm.nih.gov/pubmed/32997803 10.1002/ijc.33320 PMC7756277 32997803

[29] Teschendorff AE, Jones A, Fiegl H, Sargent A, Zhuang JJ, Kitchener HC (2012). Epigenetic variability in cells of normal cytology is associated with the risk of future morphological transformation. Genome Med.

[30] Perskvist N, Norman I, Eklund C, Litton JE, Dillner J (2013). The Swedish cervical cytology biobank: sample handling and storage process. Biopreserv Biobank..

[31] Ludvigsson JF, Almqvist C, Bonamy AK, Ljung R, Michaelsson K, Neovius M (2016). Registers of the Swedish total population and their use in medical research. Eur J Epidemiol.

[32] Hortlund M, Sundstrom K, Lamin H, Hjerpe A, Dillner J (2016). Laboratory audit as part of the quality assessment of a primary HPV-screening program. J Clin Virol.

[33] Fiegl H, Gattringer C, Widschwendter A, Schneitter A, Ramoni A, Sarlay D (2004). Methylated DNA collected by tampons: a new tool to detect endometrial cancer. Cancer Epidemiol Biomarkers Prev.

[34] Zheng SC, Webster AP, Dong D, Feber A, Graham DG, Sullivan R (2018). A novel cell-type deconvolution algorithm reveals substantial contamination by immune cells in saliva, buccal and cervix. Epigenomics.

