# Supplementary material for: DNA methylation-based detection and prediction of cervical intraepithelial neoplasia grade 3 and invasive cervical cancer with the WID™-qCIN test
Source: Clin Epigenetics. 2022 Nov 21;14:150. doi: 10.1186/s13148-022-01353-0 (PMC9682674; doi:10.1186/s13148-022-01353-0)
Supplement: Supplementary file 1 — Additional file 1: Additional figures and tables. [file 13148_2022_1353_MOESM1_ESM.pdf]

DNA methylation-based detection and prediction of cervical intraepithelial neoplasia grade 3 and invasive cervical cancer with the WID™-qCIN test

Additional File 1

Chiara Herzog*, Ph.D., Karin Sundström*, Ph.D., Allison Jones, B.Sc., Iona Evans, Ph.D., James E. Barrett, Ph.D., Jiangrong Wang, Ph.D., Elisa Redl, M.Sc., Lena Schreiberhuber, M.Sc., Laura Costas Ph.D., Sonia Paytubi Ph.D., Lukas Dostalek, Ph.D., Prof. Michal Zikan, Ph.D., Prof. David Cibula, M.D., Assoc.-Prof. Dr.P.H. Gaby Sroczynski, M.P.H., Prof. Uwe Siebert, M.D., M.P.H., M.Sc., D.Sc., Prof. Joakim Dillner[†], M.D., and Prof. Martin Widschwendter[†], M.D.

* contributed equally

[†] contributed equally

Contents

1 **Cost-effectiveness analysis** 3

2 **Table S1. Specificity and sensitivity of the WID™-qCIN for detection of CIN1, CIN2, CIN3, and invasive cancers in all samples of the LBC-CIN Diagnostic set.** 4

3 **Table S2. Specificity and sensitivity of the WID™-qCIN for detection of CIN1, CIN2, CIN3, and invasive cancers in HPV+ samples of the LBC-CIN Diagnostic set.** 5

4 **Table S3. Specificity and sensitivity of HPV subtyping (HPV16/HPV18+ versus other oncHPV) for detection of CIN1, CIN2, CIN3, and invasive cancers in HPV+ samples of the LBC-CIN Diagnostic and LBC-CIN Predictive sets.** 6

5 **Table S4. Primers used for the WID™-qCIN test.** 7

6 **Table S5. Primary screening test characteristics: sensitivity and specificity of cytology and HPV testing.** 8

7 **Table S6. Aggregated costs (per unit) for screening, diagnostic work-up, therapy, and follow-up procedures (Index year 2020) for cervical cancer.** 9

8 **Table S7. Cost effectiveness analysis: ICER results for different WID™-qCIN test costs.** 10

9 **Table S8. Cost effectiveness Analysis: ICER Results for different WID(TM)-qCIN test costs.** 11

10 **Figure S1. Flowchart of the cervical sample collection cohort from which samples for the CpG identification, LBC-CIN Discovery, LBC-CIN Diagnostic, and LBC-CIN Predictive sets were derived.** 12

11 **Figure S2. Visualisation of individual WID™-qCIN reaction values in the LBC-CIN Diagnostic Validation Set.** 14

12 **Figure S3. Flowchart of the Swedish cervical cancer screening programme.** 15

13 **References** 16

Cost-effectiveness analysis

Methods

We used a previously validated Markov-state-transition model [1] for the Austrian health care context to evaluate the long-term cost-effectiveness of implementing WID™-qCIN as a primary screening test or in adjunct to established HPV-based primary cervical cancer screening [2]. Austrian demographic, economic, clinical, and epidemiological data, along with test accuracy data from international meta-analyses for cytology and HPV testing (Table 5) as well as and original data for the WID™-qCIN (Appendix Table4) were used as model input parameters.

We adopted the Austrian healthcare payers perspective and an annual discount rate of 3% for both health effects and costs. We included direct medical costs based on actual reimbursement costs (year 2020) related to screening-related procedures, diagnostic follow-up and treatment of preinvasive and invasive cancer in Austria (aggregated costs are summarized in Table 6). For each of the different evaluated screening strategies, we determined the discounted incremental cost-effectiveness ratio (ICER) expressed as EUR per life-year gained (LYG) compared to the next non-dominated strategy under different cost assumptions for the WID™-qCIN test. The ICER expresses the costs per one LY gained, which is calculated by dividing the discounted incremental net costs between compared two alternatives by the discounted incremental net health effects between these two alternatives. Strategies are considered dominated if they provide less health benefit at higher costs when compared to any other strategy. Therefore, dominated strategies should not be considered by decision makers and no ICER is calculated. Furthermore, extended dominance is applied to eliminate strategies, for which costs and benefits are dominated by a mix of two other alternatives. We performed a threshold analysis varying the cost for WID™-qCIN to evaluate the price at which the implementation of WID™-qCIN would be cost effective in a screening setting. We used the definition of the World Health Organization (WHO) for cost-effectiveness considering health technologies as cost effective at a willingness-to-pay threshold of three times the national Gross Domestic Product (GDP) per capita (i.e., EUR 126,000 per capita in Austria for the year 2020).

As annual Pap cytology is still recommended and reimbursed for all women as of age 18 in Austria, we included annual Pap cytology screening as of age 18 years as a reference strategy. In order to compare with other screening programs in Europe, we additionally evaluated Pap cytology screening and HPV-based screening in an organized setting at screening intervals of 3 years starting at age 20 with HPV primary screening for women older than age 30. We included WID™-qCIN as a primary screening test or as a triage-test for HPV-screen-positive women. In comparison to the abovementioned strategies we assessed 1) annual WID™-qCIN primary screening starting at age 18, 2) triennial WID™-qCIN primary screening starting at age 20, 3) triennial HPV primary screening with WID™-qCIN triage starting age 30 (with Pap cytology for women age 20 to 29).

Results

Triennial WID™-qCIN was more effective in terms of life-years gained compared to the current standard annual cytology in Austria. Under a wide range of different cost assumptions for the WID™-qCIN test, primary screening with WID™-qCIN as of age 20 at triennial screening intervals was also less costly than the current standard in Austria (Tables S8 and S9). In the threshold analysis, primary screening with WID™-qCIN at triennial screening was cost effective (according to WHO criteria), if the costs of the WID™-qCIN test are is below EUR 27 per test.

In multivariate sensitivity analyses, using the lower limits of the 95% confidence intervals (95% CI) for the WID™-qCIN test sensitivity and upper 95% CI limits for the specificity, results were robust. When we used upper 95% CI limits for the WID™-qCIN test sensitivity and lower 95% CI limits for the specificity, triennial WID™-qCIN screening was cost effective when the test costs did not exceed EUR 22 (results not shown).

Table S1. Specificity and sensitivity of the WID™-qCIN for detection of CIN1, CIN2, CIN3, and invasive cancers in all samples of the LBC-CIN Diagnostic set.

	Value (95% CI)	Positive controls/ total controls	Positive cases/ total cases
**All, % (95% CI)**			
Specificity	92 (88-95)	19/239	118/267
Sensitivity (CIN1)	14 (8-21)	19/239	16/116
Sensitivity (CIN2)	55 (42-67)	19/239	36/66
Sensitivity (CIN3)	69 (56-80)	19/239	43/62
Sensitivity (Cervical cancer)*	100 (85-100)	19/239	23/23
**Age <30 years, % (95% CI)**			
Specificity	96 (89-99)	3/79	33/93
Sensitivity (CIN1)	8 (2-21)	3/79	3/39
Sensitivity (CIN2)	46 (28-66)	3/79	13/28
Sensitivity (CIN3)	59 (36-79)	3/79	13/22
Sensitivity (Cervical cancer)*	100 (40-100)	3/79	4/4
**Age ≥30 years, % (95% CI)**			
Specificity	90 (84-94)	16/160	85/174
Sensitivity (CIN1)	17 (9-27)	16/160	13/77
Sensitivity (CIN2)	61 (43-76)	16/160	23/38
Sensitivity (CIN3)	75 (59-87)	16/160	30/40
Sensitivity (Cervical cancer)*	100 (82-100)	16/160	19/19

Abbreviations: *CIN*, cervical intraepithelial neoplasia.
* includes adenocarcinoma in situ and invasive cancers.

Table S2. Specificity and sensitivity of the WID™-qCIN for detection of CIN1, CIN2, CIN3, and invasive cancers in HPV+ samples of the LBC-CIN Diagnostic set.

	Value (95% CI)	Positive controls/ total controls	Positive cases/ total cases
**All, % (95% CI)**			
Specificity	85 (73-93)	9/60	96/242
Sensitivity (CIN1)	13 (8-21)	9/60	15/112
Sensitivity (CIN2)	55 (42-67)	9/60	36/66
Sensitivity (CIN3)	69 (56-80)	9/60	42/61
Sensitivity (Cervical cancer)*	100 (29-100)	9/60	3/3
**Age <30 years, % (95% CI)**			
Specificity	100 (72-100)	0/11	30/89
Sensitivity (CIN1)	8 (2-21)	0/11	3/38
Sensitivity (CIN2)	46 (28-66)	0/11	13/28
Sensitivity (CIN3)	59 (36-79)	0/11	13/22
Sensitivity (Cervical cancer)*	100 (3-100)	0/11	1/1
**Age ≥30 years, % (95% CI)**			
Specificity	82 (68-91)	9/49	66/153
Sensitivity (CIN1)	16 (9-27)	9/49	12/74
Sensitivity (CIN2)	61 (43-76)	9/49	23/38
Sensitivity (CIN3)	74 (58-87)	9/49	29/39
Sensitivity (Cervical cancer)*	100 (16-100)	9/49	2/2

Abbreviations: *CIN*, cervical intraepithelial neoplasia.
* includes adenocarcinoma in situ and invasive cancers.

Table S3. Specificity and sensitivity of HPV subtyping (HPV16/HPV18+ versus other oncHPV) for detection of CIN1, CIN2, CIN3, and invasive cancers in HPV+ samples of the LBC-CIN Diagnostic and LBC-CIN Predictive sets.

	LBC-CIN Diagnostic	LBC-CIN Predictive
**All, % (95% CI)**		
n (controls, cases)	172, 61	124, 131
Sensitivity – % (95% CI)	59 (46–71)	56 (47–64)
Specificity – % (95% CI)	67 (60–74)	76 (67–83)
PPV – % (95% CI) †	33 (28–40)	39 (32–47)
NPV – % (95% CI) †	86 (82–89)	86 (84–89)
**Age <30 years, % (95% CI)**		
n (controls, cases)	49, 22	60, 60
Sensitivity – % (95% CI)	59 (36–79)	57 (43–69)
Specificity – % (95% CI)	76 (61–87)	67 (53–78)
PPV – % (95% CI) †	40 (29–55)	32 (25–41)
NPV – % (95% CI) †	87 (81–92)	85 (81–89)
**Age ≥30 years, % (95% CI)**		
n (controls, cases)	123, 39	64, 71
Sensitivity – % (95% CI)	59 (42–74)	55 (43–67)
Specificity – % (95% CI)	64 (55–73)	84 (73–92)
PPV – % (95% CI) †	31 (25–39)	49 (37–64)
NPV – % (95% CI) †	85 (80–89)	87 (84–90)

PPV denotes positive predictive value, **NPV** negative predictive value.
† Assumed population prevalence in the above sets: 21%

Table S4. Primers used for the WID™-qCIN test.

Target	Sequence (5'–3')	Concentration – µM
COL2A1		
Forward	TCTAACAATTATAAACTCCAACCACCAA	0.6
Reverse	GGGAAGATGGGATAGAAGGGAATAT	0.6
Probe	6-FAM-CGCCTTCATTCTAACCCAATACCTATCCCACCTCTAAA-BHQ1	0.2
DPP6		
Forward	TTATCGTAGTGTTTGTTTGTGGAAGTC	0.4
Reverse	CCCACTCCGCGCTAAACTAA	0.4
Probe	HEX-CGTGCGTCGCGCGCGTA-BHQ1	0.2
RALYL		
Forward	GCGTTTGAGAGCGGTAATATTAGTG	0.4
Reverse	CCTACTCGTCTAAACTCACAACGAAA	0.4
Probe	CY5-AGCGGTAGTTCGCGGCGAGGTT-BHQ3	0.2
GSX1		
Forward	CGTAGAGGGCGGGTTGGT	0.3
Reverse	GCGCAACACTAACGAATCCA	0.3
Probe	HEX-CGTGCGTCGCGCGCGTA-BHQ1	0.2

Table S5. Primary screening test characteristics: sensitivity and specificity of cytology and HPV testing.

Type	Sensitivity (%)	Specificity (%)
**Cytology (LSIL+) [1,2]**		
CIN1	47.1	96.7
CIN2	65.9	96.7
CIN3+	70.7	96.7
**HPV (1 pg/ml) [2]**		
CIN1	80.6	89.2
CIN2	92.6	89.2
CIN3+	96.5	89.2

References:
[1] Nanda K, McCrory DC, Myers ER, et al. Accuracy of the Papanicolaou test in screening for and follow-up of cervical cytologic abnormalities: a systematic review. *Ann Intern Med* 2000;132(10):810-9.
[2] Koliopoulos G, Nyaga VN, Santesso N, et al. Cytology versus HPV testing for cervical cancer screening in the general population. *The Cochrane Database of Systematic Reviews* 2017;8:Cd008587.

Table S6. Aggregated costs (per unit) for screening, diagnostic work-up, therapy, and follow-up procedures (Index year 2020) for cervical cancer.

Procedure	Costs in EUR (2020)
Gynaecological visit	23.02
Cervical smear Pap	5.08
Cervical smear HPV	6.84
Colposcopy	10.54
Biopsy	31.86
Histology	28.26
Pap test	7.86
HPV test	47.45
WID™-qCIN test	*
Conization	1589.00
Follow-up after conization	90.00
Therapy FIGO I	16785.00
Therapy FIGO II	62410.00
Therapy FIGO III	75025.00
Therapy FIGO IV	46678.00
Annual follow-up during years 1-3 after cancer therapy	344.00
Annual follow-up during years 4 and 5 after cancer therapy	203.00
Annual follow-up during years 6-10 after cancer therapy	132.00

* varied costs, EUR 15-35.
Abbreviations: *FIGO*, cervical cancer stage classification Fédération Internationale de Gynécologie et d'Obstétrique. *HPV*, human papillomavirus. *Pap*. Papanicolau.

Table S7. Cost effectiveness Analysis: ICER Results for different WID™-qCIN test costs.

	Cost effectiveness according to WHO criteria (ICER between 1 time and 3 times the GDP per capita) at WID™-CIN test cost of:				
	EUR 15	EUR 20	EUR 25	EUR 30	EUR 35
**Strategy**					
No Screening	---	---	---	---	---
Pap / HPV Triage: age 20y+, Int 3y, org	Dominated	Very cost effective	Very cost effective	Very cost effective	Very cost effective
WID™-qCIN, age 20yr, Int 3y, org.	Very cost effective	Very cost effective	Cost effective	Extended dominated	Extended dominated
WID™-qCIN, age 18y+, Int 1y	Dominated	Dominated	Dominated	Dominated	Dominated
Pap / HPV Triage: age 18y+, Int 1y (standard)	Dominated	Dominated	Dominated	Dominated	Dominated
HPV / WID™-qCIN Triage: age 30y; Pap: age 20-29y, Int 3y, org.	Not cost effective	Not cost effective	Not cost effective	Not cost effective	Not cost effective
HPV / Pap Triage: age 30y+; Pap: age 20-29y, Int 3y, org.	Not cost effective	Not cost effective	Not cost effective	Not cost effective	Not cost effective

Note: a strategy is very cost effective according to WHO with an ICER < 1 GDP (i.e. EUR 42,000 per capita in the year 2020) and cost effective with an ICER < 3 GDP (i.e. EUR 126,000 per capita in the year 2020).
Abbreviations: *HPV*, human papilloma virus. *Int*, interval. *org*, organized setting. *Pap*, Papanicolaou. *y*, year.

Table S8. Cost effectiveness Analysis: ICER Results for different WID™-qCIN test costs.

	ICER (in 2020 EUR per LYG) at WID™-CIN test cost of:				
	EUR 15	EUR 20	EUR 25	EUR 30	EUR 35
**Strategy**					
No Screening	---	---	---	---	---
Pap / HPV Triage: age 20y+, Int 3y, org	Dom.	10500	10500	10500	10500
WID™-qCIN, age 20yr, Int 3y, org.	9600	24200	93900	Ext. dom.	Ext. dom.
WID™-qCIN, age 18y+, Int 1y	Dom.	Dom.	Dom.	Dom.	Dom.
Pap / HPV Triage: age 18y+, Int 1y (standard)	Dom.	Dom.	Dom.	Dom.	Dom.
HPV / WID™-qCIN Triage: age 30y; Pap: age 20-29y, Int 3y, org.	269500	229600	189800	155500	160100
HPV / Pap Triage: age 30y+; Pap: age 20-29y, Int 3y, org.	393700	376500	359400	342300	325100

Abbreviations: *Dom*, dominated. *Ext. dom.*, extended dominated. *HPV*, human papilloma virus. *Int*, interval. *org*, organized setting. *Pap*, Papanicolaou. *y*, year.

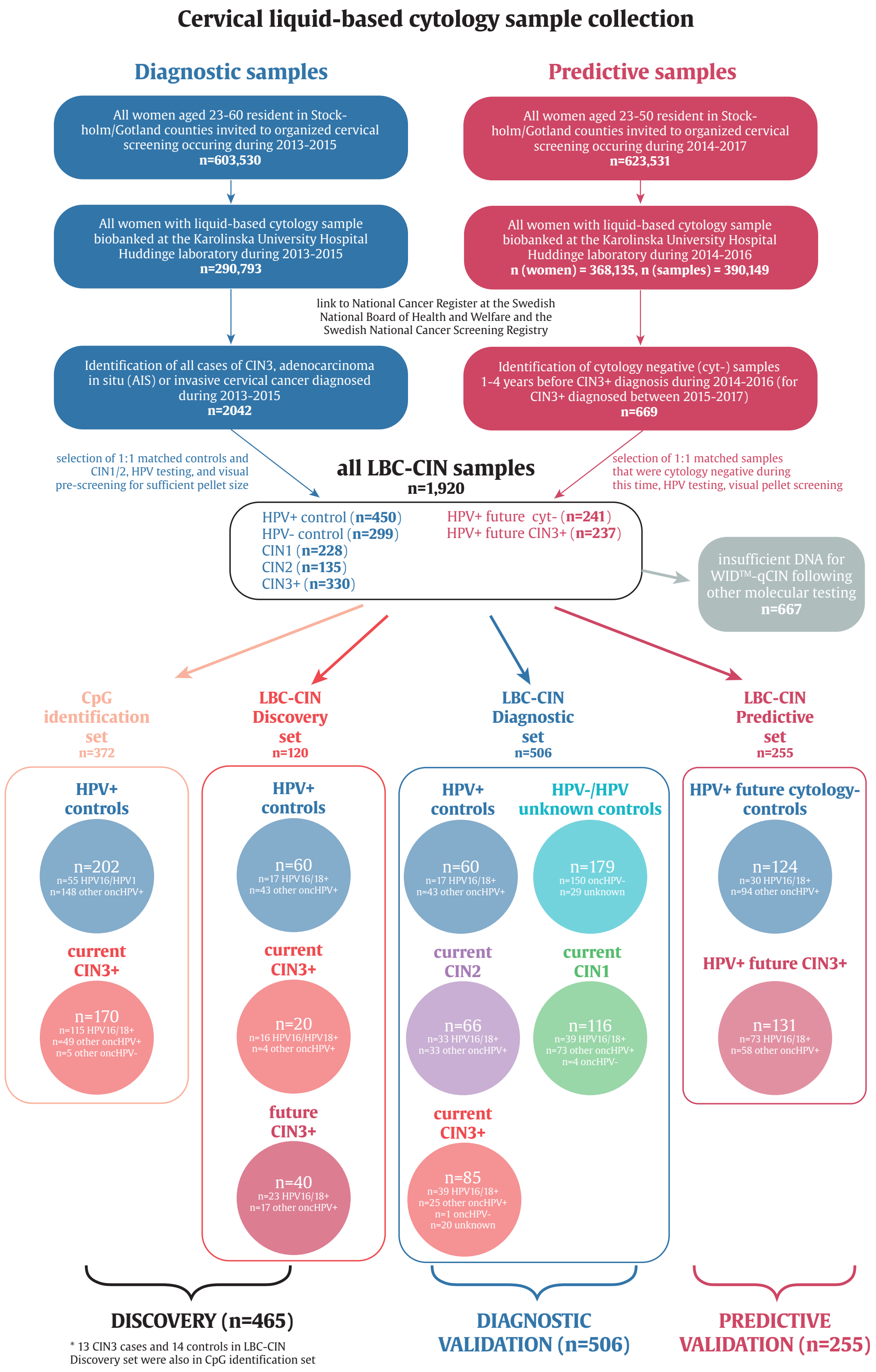

Figure S1. Flowchart of the cervical sample collection cohort from which samples for the CpG identification, LBC-CIN Discovery, LBC-CIN Diagnostic, and LBC-CIN Predictive sets were derived. For

visual screening, we blinded to case-control status and visually screened all eligible vials of biobanked samples to ensure that a visible cell pellet was present, which would indicate sufficient material for DNA extraction and subsequent molecular testing. Approximately 1/3 of samples had such a pellet that was independent of case-control or CIN3/ICC status. We subsequently aliquoted 100 μl from each sample in order for UCL to perform methylation analyses. Note: Some samples were assessed twice (i.e. in the CpG identification set for epigenome-wide discovery and in the LBC-CIN Diagnostic Validation set.

Abbreviations: **CIN** , cervical intraepithelial neoplasia. **oncHPV** , oncogenic human papillomavirus.

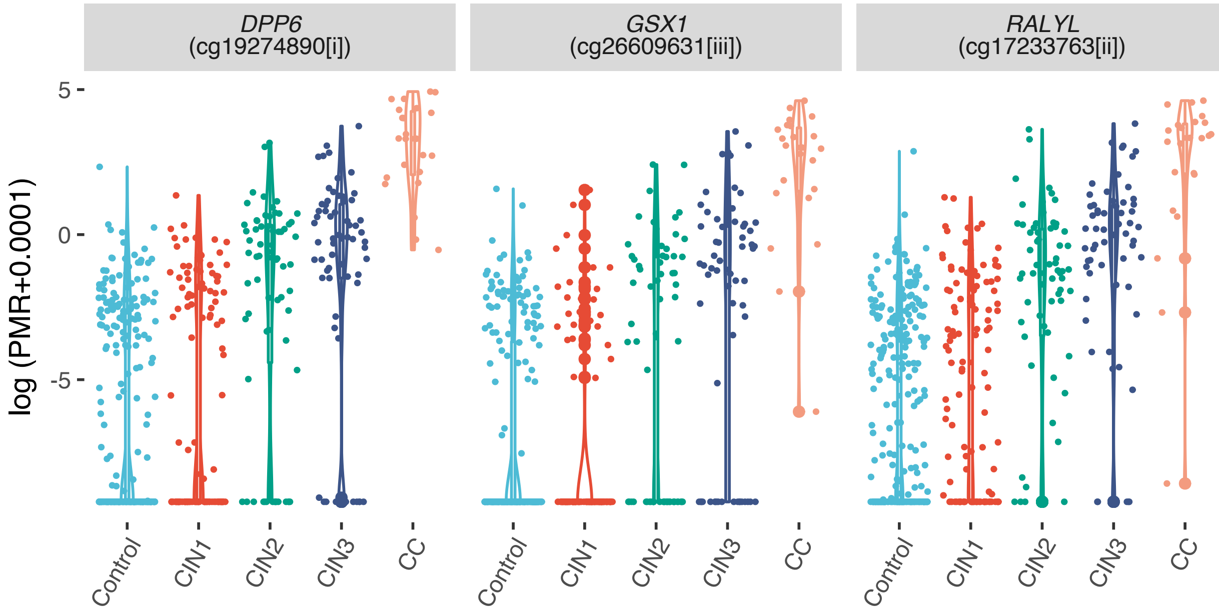

Figure S2. Visualisation of individual WID™-qCIN reaction values in the LBC-CIN Diagnostic set. Values are shown on a log scale for illustrative purposes.

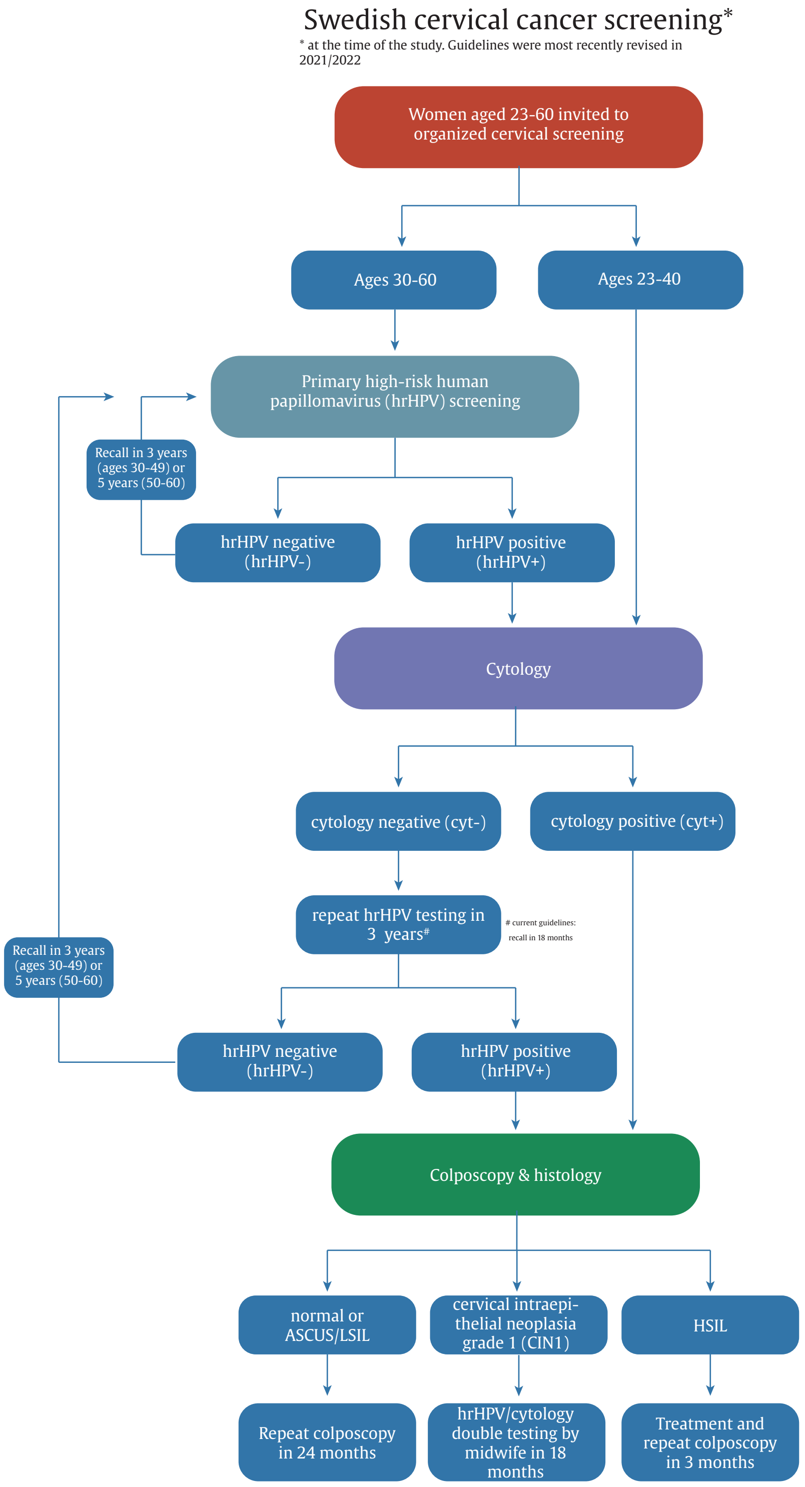

Figure S3. Flowchart of the Swedish cervical cancer screening programme. The flowchart corresponds to the guidelines at the time of the study, with most recent updates published in 2021/2022.

References

1. Siebert, U. et al. State-transition modeling: a report of the ISPOR-SMDM Modeling Good Research Practices Task Force-3. Med Decis Making 32, 690-700 (2012).
2. Sroczynski, G. et al. Reducing overtreatment associated with overdiagnosis in cervical cancer screening-A model-based benefit-harm analysis for Austria. Int J Cancer (2019) doi:10.1002/ijc.32849.
